# Near-infrared low-level laser stimulation of telocytes from human myometrium

**DOI:** 10.1007/s10103-014-1589-1

**Published:** 2014-05-29

**Authors:** Razvan-Alexandru Campeanu, Beatrice Mihaela Radu, Sanda Maria Cretoiu, Daniel Dumitru Banciu, Adela Banciu, Dragos Cretoiu, Laurentiu Mircea Popescu

**Affiliations:** 1 Department of Anatomy Animal Physiology and Biophysics, Faculty of Biology, University of Bucharest, 050095 Bucharest, Romania; 2 Neuroscience Area, International School for Advanced Studies (SISSA), 34136 Trieste, Italy; 3 Department of Neurological and Movement Sciences, University of Verona, 37134 Verona, Italy; 4 Department of Cellular and Molecular Medicine, Carol Davila University of Medicine and Pharmacy, 050474 Bucharest, Romania; 5 Department of Ultrastructural Pathology, Victor Babeş National Institute of Pathology, 050096 Bucharest, Romania; 6 Department of Molecular Medicine, Victor Babeş National Institute of Pathology, 050096 Bucharest, Romania; 7 Division of Advanced Studies, Victor Babeş National Institute of Pathology, 050096 Bucharest, Romania

**Keywords:** 1064 Nd:YAG laser, Low-level laser stimulation, Telocytes, Telopodes, Human myometrium, Pregnancy

## Abstract

Telocytes (TCs) are a brand-new cell type frequently observed in the interstitial space of many organs (see www.telocytes.com). TCs are defined by very long (tens of micrometers) and slender prolongations named telopodes. At their level, dilations—called podoms (~300 nm), alternate with podomers (80–100 nm). TCs were identified in a myometrial interstitial cell culture based on morphological criteria and by CD34 and PDGF receptor alpha (PDGFRα) immunopositivity. However, the mechanism(s) of telopodes formation and/or elongation and ramification is not known. We report here the low-level laser stimulation (LLLS) using a 1,064-nm neodymium-doped yttrium aluminum garnet (Nd:YAG) laser (with an output power of 60 mW) of the telopodal lateral extension (TLE) growth in cell culture. LLLS of TCs determines a higher growth rate of TLE in pregnant myometrium primary cultures (10.3 ± 1.0 μm/min) compared to nonpregnant ones (6.6 ± 0.9 μm/min). Acute exposure (30 min) of TCs from pregnant myometrium to 1 μM mibefradil, a selective inhibitor of T-type calcium channels, determines a significant reduction in the LLLS TLE growth rate (5.7 ± 0.8 μm/min) compared to LLLS per se in same type of samples. Meanwhile, chronic exposure (24 h) completely abolishes the LLLS TLE growth in both nonpregnant and pregnant myometria. The initial direction of TLE growth was modified by LLLS, the angle of deviation being more accentuated in TCs from human pregnant myometrium than in TCs from nonpregnant myometrium. In conclusion, TCs from pregnant myometrium are more susceptible of reacting to LLLS than those from nonpregnant myometrium. Therefore, some implications are emerging for low-level laser therapy (LLLT) in uterine regenerative medicine.

## Introduction

Thorough knowledge of the structure of the uterine wall is essential to contribute to the understanding of reproductive function. Alterations of normal function of human uterus are reported in pregnant and nonpregnant state. Often these disorders implicate the reproductive function and are difficult to manage in the absence of a specific treatment.

Telocytes (TCs) were recently described as stromal/interstitial cells found in many organs (for details, visit www.telocytes.com) including the human uterus [1, 2]. Transmission electron microscopy is considered to be the most suited method for TCs identification [3, 4]. TCs can also be identified by CD34 and PDGF receptor alpha (PDGFRα) immunohistochemistry [5–8]. The function of TCs is not well understood yet; however, evidence points towards a role of telopodes in the coordination of the surrounding cells by exosome/ectosome release [9–11]. TCs display electrical activity [12] and have been observed to form homo- and heterocellular junctions [4]. Currently, cell culture has emerged as an important research method for studying the TCs behavior [13]. Time-lapse microscopy revealed dynamically moving telopodes which were supposed to serve as guiding wires for other cells in coculture [12]. The process standing behind this dynamics of telopodes is still to be understood, and information about the biophysical properties of the telopodal plasma membrane would bring new insights.

To this purpose, we have decided to stimulate by near-infrared (NIR) laser the telopodes for testing their ability to grow and the possibility of stimulation of telopodal lateral extension (TLE) growth. The ability of TCs to form homo- and heterocellular contacts with various cell types (e.g., myocytes, immune cells, stem cells, etc.) in different organs [14–16] has a tremendous medical impact. The possibility of influencing their dynamics in vitro and in vivo by means of low-level NIR guidance can open new perspectives in uterine regenerative medicine.

The idea of optical stimulation and guidance was extensively tested on primary neuronal cell cultures or neuronal cell lines (for review, see [17–22] using low-level laser stimulation (LLLS)). Moreover, other types of cells, such as Swiss 3T3 cells, extend pseudopodia towards NIR light sources [23]. Although TCs extend long telopodes with dynamic movement [12] and are good candidates for optical stimulation by means of LLLS, the topic is still uncovered. The goal of our study was to identify, for the first time, the differences of TC response to LLLS between nonpregnant and pregnant human myometria.

## Materials and methods

### Tissue samples

Five biopsies of human myometrium were obtained from different hysterectomy specimens (benign indications) of nonmenopausal women (mean age 42.5 years). Other five specimens were obtained from the uteri of pregnant primipara women (between 38 and 40 weeks of gestation, mean age 32.5 years), at the time of cesarean section. All patients received information about the study and signed an informed consent file. All experiments have been carried out in accordance with the EU guidelines and approved by the Bioethics Committee of “Carol Davila” University of Medicine Bucharest.

### Cell cultures

Human myometrial samples were collected into sterile tubes containing Dulbecco’s modified Eagle medium (DMEM) supplemented with fetal bovine serum (FBS) 2 %, HEPES (1.5 mM), as well as 10,000 IU/ml penicillin, 0.2 mg/ml streptomycin, and 0.50 mg/ml amphotericin (Sigma Chemical), placed on ice and transported to the cell culture laboratory. Samples were processed within 30 min from surgery. Cells were cultured using the procedure described in detail elsewhere [12].

### Immunofluorescence

Immunofluorescent staining was performed on cells cultured on coverslips, at fourth passage. The cells were fixed in 2 % paraformaldehyde for 10 min, washed in phosphate-buffered saline (PBS), and then incubated in PBS containing 1 % bovine serum albumin (BSA) for another 10 min. Cells were washed again and permeabilized in PBS containing 0.075 % saponin for 10 min (all reagents were from Sigma Chemical, St. Louis, MO, USA). Incubation with the primary antibodies was performed for 1 h, at room temperature, using antihuman antibodies: CD34, goat polyclonal (sc-7045), 1:50 (Santa Cruz Biotechnology, Inc., Heidelberg, Germany), and PDGFRα rabbit polyclonal (sc-338), 1:100 (Santa Cruz Biotechnology, Inc., Heidelberg, Germany). After three serial rinses, the bound primary antibodies were detected with secondary donkey anti-goat antibody conjugated to Alexa Fluor 546, 1:250, and goat anti-rabbit antibody conjugated to Alexa Fluor 488, 1:250; all were from Invitrogen Molecular Probes, Eugene, OR, USA. Nuclei were finally counterstained with 1 μg/ml 4′,6-diamidino-2-phenylindole (DAPI) (Sigma-Aldrich).

Negative controls were obtained following the same protocol, but omitting the primary antibodies. Samples were examined under a Nikon TE300 microscope equipped with a Nikon DS-Qi1 camera, Nikon PlanApo ×20 and ×40 objectives, and the appropriate fluorescence filters.

### Near-infrared low-level laser stimulation

The optical stimulation of the TLE growth was done by means of a MicroTweezers Module twinflex Rel. 4.2 system (Carl Zeiss, Germany) mounted on an inverted microscope AxioObserver D1 (Carl Zeiss, Germany). We used a diode-pumped solid-state IR neodymium-doped yttrium aluminum garnet (Nd:YAG) laser (Ventus 1064-3000, Carl Zeiss), continuous wave (cw) mode, wavelength 1,064 nm, power 3,000 mW, transverse mode TEM_00_, beam divergence <1 mrad, beam diameter 2.5 mm. The parameters of the laser beam (e.g., output power, spot size, position) were controlled by the RoboSoftware 4.3 Pro SP2 (Carl Zeiss, Germany). The beam was focused on the cells through a Plan-Neofluar ×100/1.3 oil objective. During telopodal stimulation, the laser output power was set to 60 mW, and the spot size was 2 μm. The laser spot size was controlled and done by optically de-focusing the beam to rich the desired size, similar to [17]. The beam was applied on the telopode surface as pulses of 1 s length with a frequency of 0.1 Hz for appropriate periods of stimulation. The whole optical setup is placed on top of a vibration-isolated table (Thorlabs, USA).

The experiments of optical stimulation of TCs consist in exposing a viable telopode to a laser beam (as described above) by placing approximately half of the laser beam on a TLE. The laser beam position is continuously adjusted as the telopode expands laterally. The TLE growing speed has been calculated from the moment it started to grow after being stimulated with the laser to the moment it stopped growing and started to retract; considering the growing distance in a given time, the average growing speed was estimated.

TCs were continuously perfused using a MPS-2 multichannel perfusion system with a micromanifold of 100 μm (World Precision Instruments, USA) at a rate flow of 1 μl/s. In the acute experiment, LLLS is performed before (control) and after 30 min of mibefradil (1 μM) (from Sigma-Aldrich, St. Louis, MO, USA) continuous perfusion on two different telopodes of the same TC. TCs were chronically (24 h) exposed to mibefradil (1 μM) by overnight incubation at 37 °C in a humidified atmosphere (5 % CO_2_ in air), and LLLS was performed in the next day. In both types of treatments, mibefradil is prepared into DMEM supplemented with FBS 10 %.

### Statistical analysis

Data are analyzed and plotted using Excel (Microsoft, Redmond, WA, USA). The values of growth rate are reported as mean ± SD. Unpaired Student’s *t* test was employed to compare the growth rates upon LLLS on TCs from nonpregnant vs. pregnant myometrium. Meanwhile, paired Student’s *t* test was used to compare growth rates upon LLLS on TCs from pregnant myometrium before and after mibefradil treatment.

## Results

**Fig. 1:**
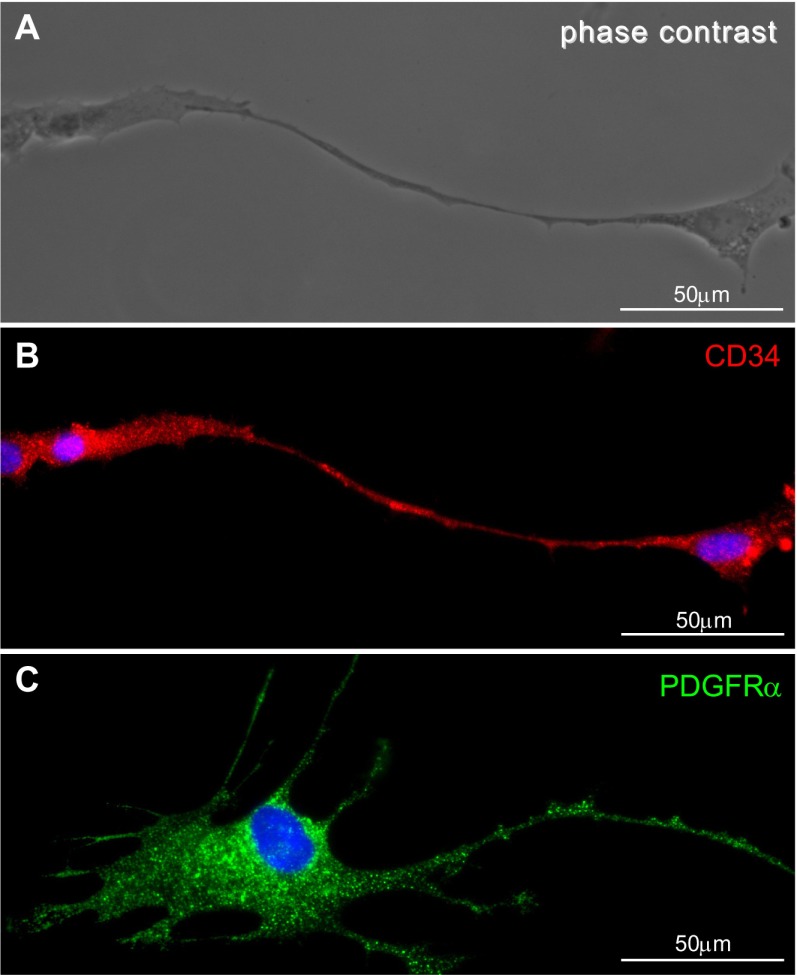
TCs in myometrial cell culture (fourth passage, day 3). **a** Phase contrast microscopy of typical a TC with very long telopodes. **b** Distribution of CD 34 immunopositivity in the same TC. **c** Cells that display the TC morphology express PDGFRα. *Scale bar* = 50 μm

In this study, we identified TCs in myometrial cell culture, using accepted criteria: morphology under phase contrast microscopy and immunocytochemistry criteria in fluorescence microscopy. TCs were seen as cells with very long telopodes in phase contrast microscopy (Fig. 1a). In fluorescence microscopy, CD34-positive cells were seen (Fig. 1b) having approximately the same morphology with PDGFRα-positive cells (Fig. 1c).

The mean duration of TLE stimulation was around 30 min or above, and it was chosen depending on the moment TLE stopped growing or retraction. The period of TLE exposure to LLLS and the beam laser characteristics are comparable to the previous reports on in vitro neuronal stimulation [17, 18].

**Fig. 2:**
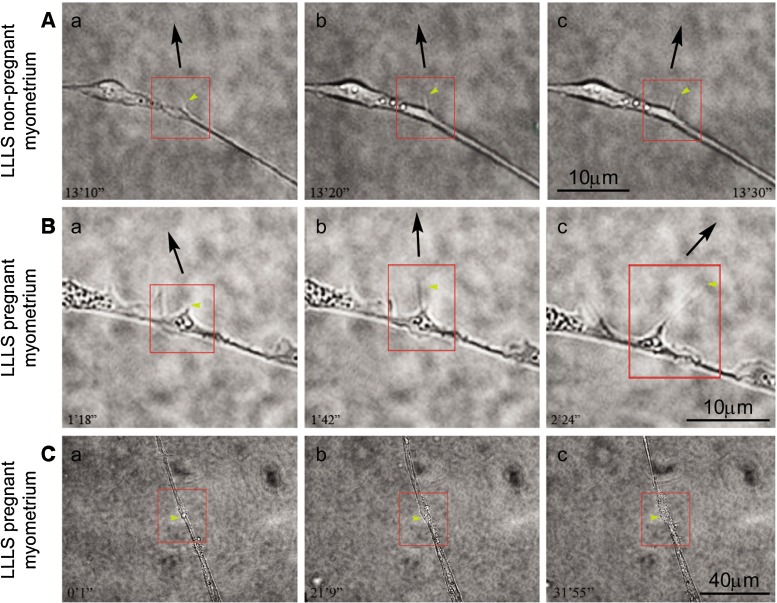
Comparative LLLS effect on TCs from nonpregnant and pregnant myometrium cell cultures (fourth passage, day 3). **a** TLE growth of TCs from nonpregnant myometrium. The angle of the TLE deviation is ≤30°. The time course of this effect is 20 s. **b** TLE growth of TCs from pregnant myometrium. The angle of the TLE deviation is between 30° and 72°, while the time course of this effect is longer—1 min and 6 s. *Scale bar* = 10 μm. **c** Telopodal local thickening upon optical tweezer stimulation was obtained in 25 % of TCs from pregnant myometrial cell culture. *Scale bar* = 40 μm. *Yellow arrows* indicate the TLE subjected to LLLS. The *black arrows* indicate the direction of the TLE. *Each red square* evidences the region of interest for the LLLS effect

Reactivity of telopodes to LLLS varies in pregnant and nonpregnant myometrium. There is a net difference between the reactivities to optical stimulation of telopodes originating from pregnant (Fig. 2b(a–c)) or nonpregnant uterus (Fig. 2a(a–c)). Telopodes from pregnant uterus are more prone to extend upon LLLS compared to those from nonpregnant uterus. The maximal length of TLE upon LLLS in telopodes from pregnant myometrium was 7.4 μm, while only a maximal growth of 2.2 μm was attained in telopodes from pregnant myometrium. It should be also taken into account the difficulty to obtain an LLLS TLE growth in TCs from nonpregnant myometrium, the required time of stimulation sometimes being three times higher than that in pregnant myometrium.

**Fig. 3:**
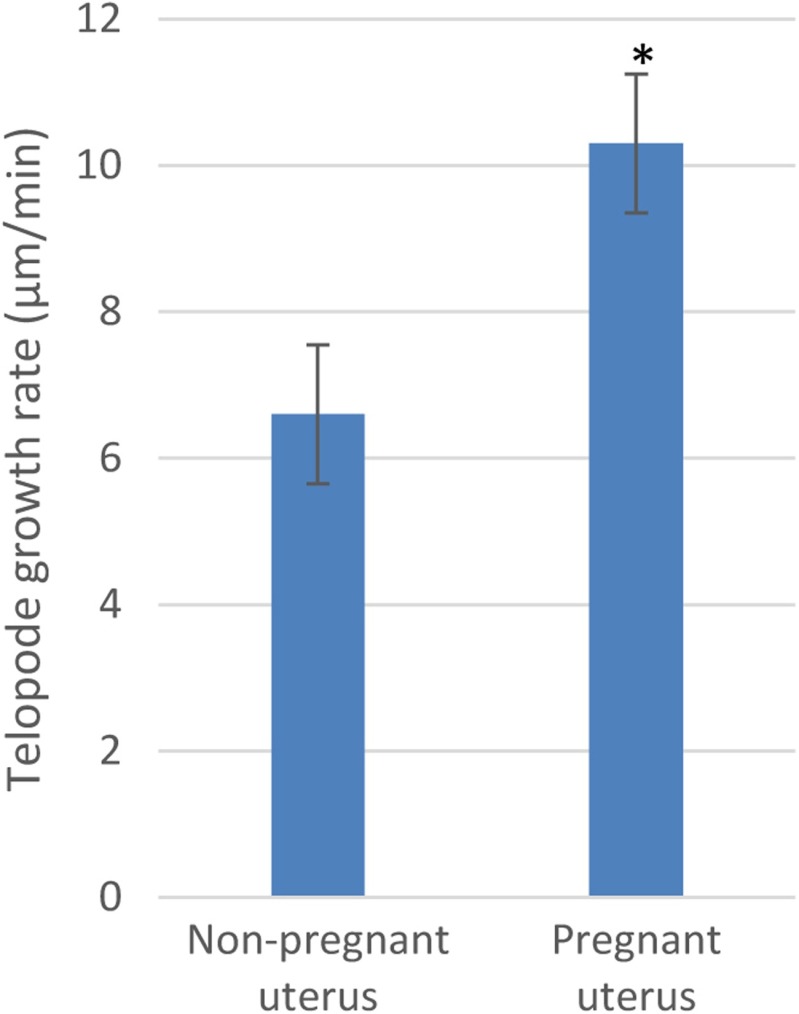
The comparative average of telopodal growth rate upon LLLS between TCs from nonpregnant and pregnant myometrium. *p < 0.01, unpaired Student’s *t* test

We have found a speed rate of TLE growth of 10.3 ± 1.0 μm/min (*n* = 6) in TCs from pregnant myometrium, which is significantly higher than 6.6 ± 0.9 μm/min (*n* = 5) in TCs from nonpregnant myometrium, *p* < 0.01, unpaired Student’s *t* test (Fig. 3).

In both preparations, telopodes seem to accumulate a big part of their resources near the stimulation area, and the laser beam with finger-like structures was probed. In some experiments, the telopode looks like it is breaking off its old connection, maintaining only thin “anchors” beyond the point of stimulation.

We tested whether we could deviate by LLLS the direction of TLE growth by at least 30° away from the original direction of TC growth, following a protocol previously described on NG108 neuroblastoma cell line [24]. The black arrows in Fig. 2a, b indicate the direction of the TLE. As we are working on human tissue, it was impossible to accumulate a large number of data as in previous studies on neuronal guidance. We have obtained a deviation below or up to 30° in the TCs from nonpregnant myometrium (Fig. 2a), while a deviation above 30° was attained in one preparation or even 72° in TCs from pregnant myometrium (Fig. 2b).

Twenty-five percent of TCs from pregnant uterus present local thickening of the telopode upon LLLS (Fig. 2c(a–c)). The local thickening phenomenon was directly correlated with a delayed telopodal response to stimulation (>1,000 s). The great variability of response to LLLS in pregnant myometrium must be considered as very important, probably being correlated with distinct uterine morphological characteristics in each patient.

We found that mibefradil modulates LLLS effect on TLE from pregnant myometrium. It is already known that mibefradil inhibits the bioelectrical signal and uterine contractile forces [25], and we have tested the combined effect of mibefradil and LLLS on TCs. TCs from pregnant myometrium have been exposed to mibefradil (1 μM), a selective antagonist of T-type calcium channels [26].

**Fig. 4:**
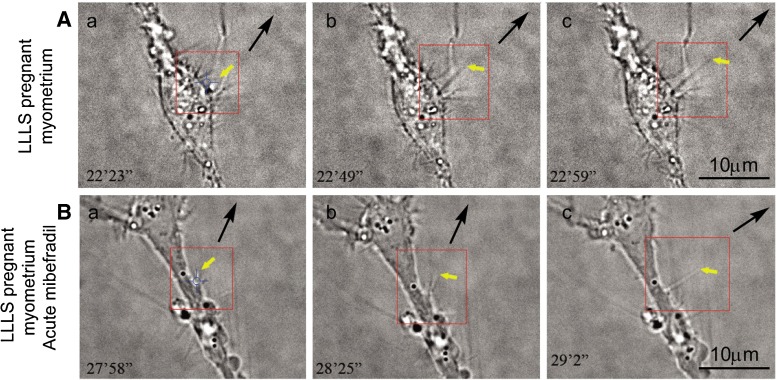
Mibefradil effect on TLE upon LLLS in pregnant myometrium (myometrial interstitial cell culture at fourth passage, day 3). **a** Untreated TCs exposed to LLLS were considered as control. The time course of LLLS effect in these images (*a*–*c*) is 36 s. We can observe how a TLE grows (*yellow arrow*). **b** Mibefradil (1 μM) was perfused for 30 min, and afterwards, TCs were re-exposed to LLLS. Comparison of the TLE growth rate reveals that in 1 min and 4 s, the length of TLE is approximately the same as that in control and that the angle of the deviation is slightly above 30°. *Yellow arrows* indicate the TLE subjected to LLLS. The *black arrows* indicate the direction of the TLE. *Each red square* evidences the region of interest for the LLLS effect. *Scale bar* = 10 μm

Acute (30 min) and chronic (24 h) exposure to mibefradil was done, and the LLLS effect on TLE growth rate was measured. In pregnant myometrium, the LLLS effect was tested on TCs per se (control; Fig. 4a(a–c)) and on TCs exposed to mibefradil (1 μM; Fig. 4b(a–c)).

**Fig. 5:**
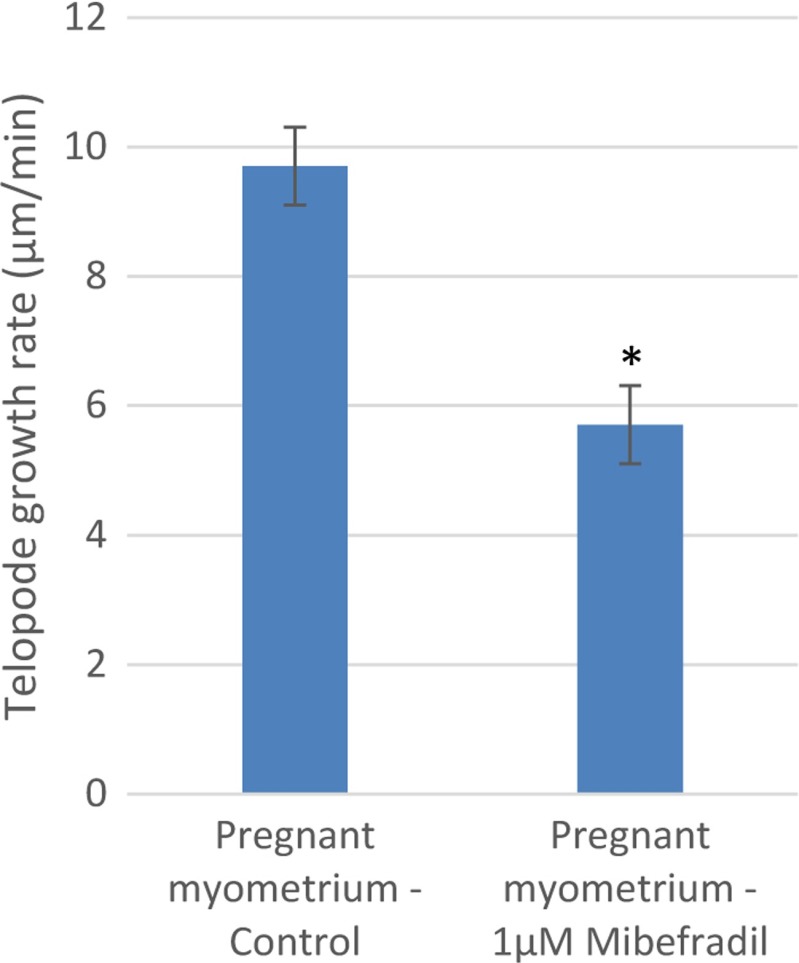
The comparative average growth rate of TLE upon LLLS between TCs from pregnant myometrium before and after acute mibefradil (30 min) treatment. *p < 0.05, paired Student’s *t* test

The chronic exposure to mibefradil determined a decrease in the growth rate, 5.7 ± 0.8 μm/min (*n* = 3), that is significantly lower than that in control conditions (9.7 ± 0.4 μm/min, *n* = 3; *p* < 0.05, paired Student’s *t* test; Fig. 5). The control value of the growth rate for pregnant myometrium was found to be different from the above-reported values. It should be noted that the LLLS growth rate after acute mibefradil treatment in pregnant myometrium is below the growth rate for control nonpregnant myometrium (*p* < 0.05, unpaired Student’s *t* test). After chronic exposure to mibefradil, LLLS performed on TCs from pregnant myometrium indicated an inhibition of the growth process.

The LLLS-induced deviation in telopodal growth direction was also monitored. Acute mibefradil treatment accentuates the angle of deviation above 30° (Fig. 4b). However, due to the large variability of responses of the TCs from pregnant myometrium to LLLS, it is difficult to estimate the exact increase in the angle of deviation due to acute mibefradil exposure.

The same experiment was performed on TCs from nonpregnant myometrium, and both acute and chronic mibefradil exposures completely abolished the TLE growth rate upon LLLS.

## Discussion

This study provides evidence for the presence of TCs in myometrial interstitial cell cultures, identified by their morphology and CD34 and PDGFRα positivity. Our findings are in correlation with recent data suggesting that PDGFRα-positive and CD34-positive cells are the same cell type—the TCs [6–8].

Low-level laser therapy (LLLT) has an extensive medical use, and the idea of using sub-thermal doses of laser light dated from the early 1970s (see reviews [27, 28]). Although the medical use of high power NIR lasers in endometrial laser intrauterine thermotherapy [29, 30] or endometrial resection and ablation [31, 32] is a clinical routine, uterine LLLT interventions are still pioneering. Therefore, our data on cellular mechanisms underlying in vitro LLLS of TCs are pushing forward this domain.

The differences in TC reactivity to LLLS highlighted by our study in human nonpregnant and pregnant myometrium are not surprising since previous morphological studies have proved significant differences in telopodal width and in podomic thickness and gauge in human nonpregnant and pregnant myometrium [12]. A possible explanation of TC differences in reactivity to LLLS might be related to distinct cytoskeleton characteristics of TCs in pregnant uterus. A recent study indicates that the expression of integrins (ITGA5, ITGA7, ITGAV, and ITGB3) increases in pregnant myometrium, and there is important colocalization with focal adhesion proteins in human myometrium at term, and it was also emphasized that mechanical signals are transmitted from the extracellular matrix through focal adhesions in pregnant human myometrium [33].

Frequency domain NIR spectroscopy has proved that optical properties of the human uterine cervix are influenced by the hormonal status depending on the phases of menstrual cycle [34] or by pregnancy [35]. Therefore, we might suppose that the uterine remodeling in pregnancy is correlated with changes in cellular dynamics and morphology, and TCs are actively participating in this 3D network reorganization. All these optical properties can be distinctly exploited in LLLS on human pregnant and nonpregnant myometrium.

It was suggested that the TC network may be involved in mechanical modulations and remodeling in various organs [36]. In particular, this issue is very interesting in the uterus, as the mechanical modulation exerted by TCs on other cell types can be distinct in pregnant and nonpregnant myometrium. The differences founded in TC reactivity to LLLS can be related to extensive changes in cell-cell communication residing in exosomes trafficking through the telopodes. A very recent challenging idea considers the cross talk between TCs-exosomes-gap junctions-cytoskeleton to be the equivalent of the primitive nervous system [11]. Cytoskeleton elements have had also being implicated as a substrate responsible for guidance of neuronal growth upon optical stimulation [19]. Telopodes also contain cytoskeleton elements, proved very recently, when the cellular TC proteome was analyzed and revealed the presence of proteins from intermediate filaments (56 %), actin cytoskeleton (19 %), and microtubule (6 %) [37]. The higher growth plasticity of the telopodes with respect to the neuronal growth cone should be taken into account since this parallelism can be further exploited, as the growth rate reported in our study is significantly higher than the previous data on neuronal optical guidance [21, 22].

The contribution of TCs in mechanical stretching during uterine contraction is still to be understood; nevertheless, membrane stiffness properties tested by LLLS can reveal interesting molecular mechanisms. It is of particular interest the fact that the telopodes of TCs from nonpregnant myometrium are intensively positive for vimentin [38], a cytoskeleton protein. A recent study proved that vimentin is very important in cytoplasmic stiffness of optically trapped mouse embryonic fibroblasts using a 1,064-nm Nd:YAG laser at a power of 200 mW [39]. The differences that we observed between pregnant and nonpregnant myometrium can possibly be explained in terms of telopodal stiffness due to vimentin-based anchoring mechanisms. Additionally, TCs from the human myometrium are positive for PDGFR, and PDGFR signaling might also modulate the telopodal membrane mechanical properties, in an analog manner, as described in vascular smooth muscle cells [40].

In conclusion, taking into consideration the profound physiological myometrial remodeling during pregnancy, which involves extensive structural and molecular changes, our findings suggest that the molecular mechanisms activated in TCs by means of LLLS are distinct in human nonpregnant and pregnant myometrium. Therefore, LLLT in human uterus should consider these cellular differences in pregnant and nonpregnant myometrium, and the laser power and exposure time should be adequately chosen.
